# The nomogram model predicts relapse risk in myelin oligodendrocyte glycoprotein antibody-associated disease: a single-center study

**DOI:** 10.3389/fimmu.2025.1527057

**Published:** 2025-03-03

**Authors:** Jiafei Cheng, Zhuoran Wang, Jing Wang, Xiaomin Pang, Jianli Wang, Meini Zhang, Junhong Guo, Huaxing Meng

**Affiliations:** ^1^ Department of Neurology, First Hospital of Shanxi Medical University, Taiyuan, Shanxi, China; ^2^ Department of First Clinical Medical College, Shanxi Medical University, Taiyuan, Shanxi, China

**Keywords:** myelin oligodendrocyte glycoprotein, myelin oligodendrocyte glycoprotein antibody-associated disorder, relapse prediction, nomogram, risk factors

## Abstract

**Background:**

Myelin oligodendrocyte glycoprotein antibody-associated disease (MOGAD) is an autoimmune disorder of the central nervous system, characterized by seropositive MOG antibodies. MOGAD can present with a monophasic or relapsing course, where repeated relapses may lead to a worse prognosis and increased disability. Currently, little is known about the risk factors for predicting MOGAD relapse in a short period, and few established prediction models exist, posing a challenge to timely and personalized clinical diagnosis and treatment.

**Methods:**

From April 2018 to December 2023, we enrolled 88 patients diagnosed with MOGAD at the First Hospital of Shanxi Medical University and collected basic clinical data. The data were randomly divided into a training cohort (80%) and a validation cohort (20%). Univariate logistic regression, least absolute shrinkage and selection operator (LASSO) regression and multivariate logistic regression were used to identify independent risk factors for 1-year relapse. A prediction model was constructed, and a nomogram was developed. The receiver operating characteristic (ROC) curve, calibration curve, and decision curve analysis (DCA) were used to evaluate and internally validate model performance.

**Results:**

Among 88 MOGAD patients, 29 relapsed within 1 year of onset (33%). A total of 4 independent risk factors for predicting relapse were identified: female sex (*P*=0.040), cortical encephalitis phenotype (*P*=0.032), serum MOG antibody titer ≥1:32 (*P*=0.007), and immunosuppressive therapy after the first onset (*P*= 0.045). The area under curve (AUC) value of the nomogram prediction model constructed with these four factors was 0.866 in the training cohort, and 0.864 in the validation cohort. The cutoff value of the total nomogram score was 140 points, distinguishing the low relapse risk group from the high relapse risk group (*P* < 0.001). The calibration curve demonstrated high consistency in prediction, and the DCA showed excellent net benefit in the prediction model. Tested by ROC curve, calibration curve, and DCA, the nomogram model also demonstrates significant value in predicting MOGAD relapse within 2 years.

**Conclusion:**

The nomogram model we developed can help accurately predict the relapse risk of MOGAD patients within one year of onset and assist clinicians in making treatment decisions to reduce the chance of relapse.

## Introduction

Myelin oligodendrocyte glycoprotein (MOG), a glycoprotein located on the outermost myelin layer in oligodendrocyte (1), participates in myelin sheath integrity, adhesion, and cell surface interaction (2). In humans, the MOG is expressed exclusively in the central nervous system (CNS) (3). MOG antibody-associated disease (MOGAD) is a new entity in the spectrum of demyelinating diseases proposed in 2018 (4). Its clinical course, immunological features, imaging findings, and response to treatment are different from anti-aquaporin-4 (AQP4) antibody seropositive neuromyelitis optica spectrum disorders (NMOSD) and classical multiple sclerosis (MS). The clinical phenotypes of MOGAD are diverse, including optic neuritis (ON), myelitis, acute disseminated encephalomyelitis (ADEM), cerebral monofocal or polyfocal deficits, brainstem or cerebellar deficits, and cerebral cortical encephalitis (CCE). In addition, benign intracranial hypertension can also serve as a rare phenotype of MOGAD (5).

MOGAD typically presents with a relapsing and remitting course, with approximately 35%-60% of cases at risk of relapse (6). The disability associated with the MOGAD varies among individuals, some patients may experience severe neurological impairment, while others may have a better recovery. Overall, MOGAD has a better prognosis compared to NMOSD, but repeated relapses can cause cumulative disability (7). Although previous studies have attempted to predict the relapse of MOGAD, the conclusions have been inconsistent (8–11). There is relatively rare involvement in constructing predictive models for MOGAD relapse. Therefore, understanding the risk factors for relapse and prognosis is crucial for clinical management.

In our study, we aimed to identify the independent risk factors for MOGAD relapse and establish a nomogram-based clinical prediction model, to provide a convenient, fast, and visual quantitative score for MOGAD relapse risk, thereby assisting clinicians in making decisions on MOGAD treatment and follow-up.

## Methods

### Participants

This data was from a single-center in China multicenter neuro-inflammatory diseases registry of Neuro-Inflammatory Diseases (CNRID, NCT05154370), an observational (noninterventional) multicenter cohort study to collect clinical information from patients who provided informed consent.

**Figure 1:**
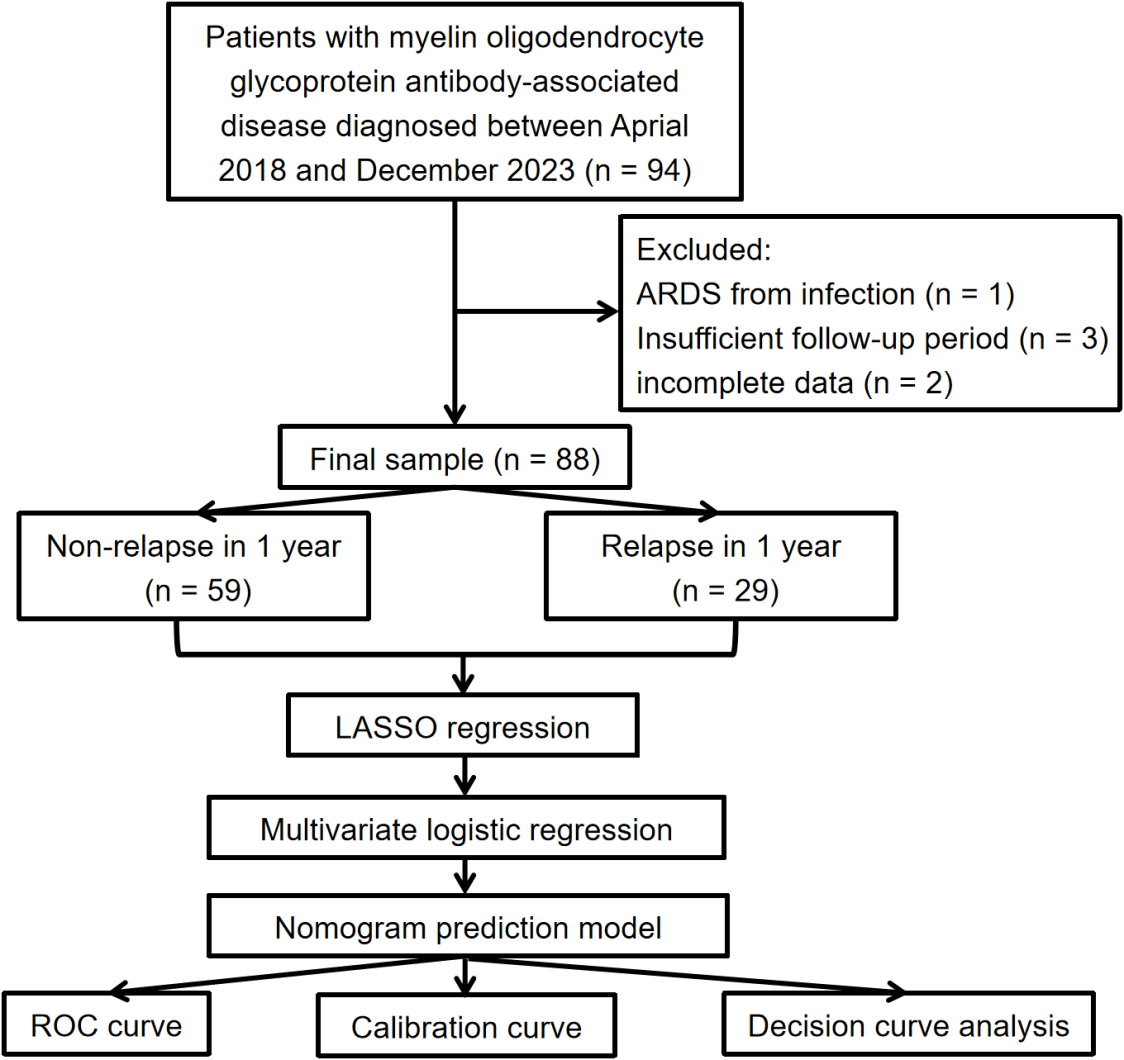
Flowchart of patient selection, grouping and statistical analysis in this study.

The inclusion criteria were based on the latest diagnostic recommendations made by the international MOGAD expert group, requiring patients to have one core clinical phenotype (ON, myelitis, ADEM, cerebral monofocal or polyfocal deficits, brainstem or cerebellar deficits, or cerebral cortical encephalitis) and clear positive serum MOG antibodies (12). If the antibody titer was unavailable or the result was low positive, diagnosing MOGAD required at least one clinical or MRI supportive feature. Exclusion criteria included: 1) Follow-up after diagnosis of less than one year; 2) Presence of other autoimmune diseases, infectious diseases, or severe mental illness; 3) Incomplete clinical information. From April 2018 to December 2023, 94 patients were initially diagnosed with MOGAD at the First Hospital of Shanxi Medical University. Of these, 6 patients were excluded: 1 patient due to ARDS from infection, 3 patients had a follow-up period of less than 12 months, and 2 patients had incomplete information. Ultimately, 88 patients were included in the follow-up study. The patient selection flow chart, grouping, and data analysis process are presented in **Figure 1**.

### Outcome measures

The primary outcome of this study was to observe and document the course and disease status of MOGAD patients at least one year after diagnosis and intervention. The disease course was categorized as either monophasic or relapse. Relapse was defined as the onset of new neurological deficits or the worsening of previously stable/improved neurological function, in the absence of fever or infection, occurring at least 30 days after the previous clinical demyelinating event, with symptoms lasting at least 24 hours (12).

### Data collection

A total of 21 characteristics of MOGAD patients in the relapse and non-relapse groups were collected within 1 year of onset, with general information (including the age of onset and gender), clinical information (including phenotype, allergy history, the time interval from initial onset to first diagnosis, treatment methods during acute and maintenance phases), laboratory test results (cerebrospinal fluid tests and serum MOG antibody titers (measured by fixed cell-based assays (CBA))), and the severity of neurological impairment (assessed using the Expanded Disability Status Scale (EDSS)) included. These indicators were intended to provide a comprehensive assessment of the potential risk of disease relapse.

### Statistical analysis

SPSS (V27.0) and R (4.3.2) were utilized for statistical analysis and graph generation. Categorical variables were reported as numbers (percentages), and differences between groups were evaluated using the chi-square test. Continuous variables were presented as mean ± standard deviation (SD), and group differences were assessed using Student’s t-test. The Shapiro-Wilk test assessed the normality of the data.

To develop and validate the nomogram, we randomly divided the entire dataset into a training cohort (80% of the data) and a validation cohort (20% of the data). Univariate logistic regression analysis compared differences between groups, and Data were presented as odds ratio (OR) with 95% confidence interval (CI). The least absolute shrinkage and selection operator (LASSO) regression was employed to screen potential risk factors for predicting 1-year relapse. LASSO regression screens variables by shrinking coefficient estimates to zero, with the degree of shrinkage dependent on an additional parameter λ. To determine the optimal value of λ, ten cross-validations were used to select λ by the minimum criterion. The four potential predictors selected by LASSO regression were included in the multivariate logistic regression to determine independent risk factors for relapse within one year of MOGAD onset and to construct a nomogram score prediction model.

The receiver operating characteristic (ROC) curve and area under the curve (AUC) were used to evaluate the predictive discrimination ability of the nomogram model. The optimal cutoff point of the nomogram model was determined by maximizing the Youden index (sensitivity+specificity-1). A calibration curve was drawn to evaluate the predictive ability of the nomogram. Decision curve analysis (DCA) assessed the clinical net benefit of the model. *P* < 0.05 was considered to be statistical significance.

## Results

### General characteristics of MOGAD patients

A total of 88 MOGAD patients met the study’s inclusion and exclusion criteria. The demographic, clinical, and laboratory characteristics at the first onset are presented in **Table 1**. The cohort comprised 46 females (52.3%) and 42 males (47.7%). The age of onset ranged from 6 to 71 years, with a mean age of 37.3 ± 16.0 years. The mean time from onset to diagnosis was 10.0 ± 29.5 months. The most common first-onset phenotype was ON (43.2%), followed by myelitis (33.0%), Brainstem or cerebellar deficits (14.8%), CCE (13.6%), cerebral monofocal or polyfocal deficits (6.8%), and ADEM (4.5%). A minority of patients (14.8%) exhibited mixed phenotypes at the first onset. Cerebrospinal fluid (CSF) was tested, and serum MOG-IgG and autoimmune antibodies were detected. The mean CSF pressure was 174.0 ± 40.3 mmH_2_O, the mean CSF white blood cell count was 44.0 ± 48.7 × 10^6/L, and the median CSF protein level was 0.86 ± 1.29 g/L. Among these, 52 patients (59.1%) had serum MOG antibody titer ≥ 1:32, and 36 patients (40.9%) tested positive for other serum autoimmune antibodies. The treatment of MOGAD is divided into two phases: acute phase treatment and maintenance therapy in the remission phase. In the acute phase, high-dose corticosteroid pulse therapy is the most commonly used treatment (53.4%), followed by high-dose corticosteroid pulse therapy combined with intravenous immunoglobulin (IVIg) (20.5%), IVIg alone (6.8%), and inadequate treatment (20.5%). In the remission phase, the most common treatment is oral corticosteroids (51.1%), followed by oral corticosteroids combined with mycophenolate mofetil (MMF) (13.6%) and oral corticosteroids combined with rituximab (RTX) (12.5%). Inadequate treatment occurs in 22.7% of cases. The mean EDSS score was 3.0 ± 1.5 at the first onset and 2.1 ± 4.5 at the last follow-up. The mean EDSS improvement rate post-treatment was 31.6 ± 35.9%.

**Table 1:** Baseline characteristics of MOGAD patients.

Variables	All patients	Non-relapse in 1 year	Relapse in 1 year	*P* value
	(N = 88)	(N=59)	(N=29)	
Sex, n (%)				
Female	42 (47.7)	25 (42.4)	21 (72.4)	0.010*
Male	46 (52.3)	34 (57.6)	8 (27.6)	
Age at onset, mean ± sd, year	37.3 ± 16.0	37.4 ± 15.2	37.2 ± 17.8	0.956
Time from onset to diagnosis, mean ± sd, month	10.0 ± 29.5	9.4 ± 31.8	11.4 ± 24.7	0.769
Allergic history, n (%)				
Yes	5 (5.7)	2 (3.4)	3 (10.3)	0.207
No	83 (94.3)	57 (96.6)	26 (89.7)	
Optic neuritis, n (%)				
Yes	38 (43.2)	24 (40.7)	14 (48.3)	0.499
No	50 (56.8)	35 (59.3)	15(51.7)	
Myelitis, n (%)				
Yes	29 (33.0)	23 (39.0)	6 (20.7)	0.091
No	59 (67.0)	36 (61.0)	23 (79.31)	
ADEM, n (%)				
Yes	4 (4.5)	2 (3.4)	2 (6.9)	0.467
No	84 (95.5)	57 (96.6)	27 (93.1)	
Brainstem or cerebellar deficit, n (%)				
Yes	13 (14.8)	9 (15.3)	4 (13.8)	0.856
No	75 (85.2)	50 (84.7)	25 (86.2)	
Cerebral monofocal or polyfocal deficit, n (%)				
Yes	6 (6.8)	5 (8.5)	1 (3.4)	0.395
No	82 (93.2)	54 (91.5)	28 (96.6)	
Cerebral cortical encephalitis, n (%)				
Yes	12 (13.6)	4 (6.8)	8 (27.6)	0.013*
No	76 (86.4)	55 (93.2)	21 (72.4)	
Mixed phenotype, n (%)				
Yes	13 (14.8)	7 (11.9)	6 (20.7)	0.278
No	75 (85.2)	52 (88.1)	23 (79.3)	
CSF pressure, mean ± sd, mmH_2_O	174.0 ± 40.3	171.9 ± 38.6	178.2 ± 44.0	0.494
CSF leucocyte count, mean ± sd, 10^6^/L	44.0 ± 48.7	45.2 ± 51.4	41.6 ± 43.6	0.745
CSF protein level, mean ± sd, g/L	0.86 ± 1.29	1.0 ± 1.5	0.6 ± 0.4	0.125
Serum MOG antibody titer, n (%)				
≥ 1:32	52 (59.1)	29 (49.2)	23 (79.3)	0.009**
< 1:32	36 (40.9)	30 (50.8)	6 (20.7)	
Serum concomitant autoantibodies^#^, n (%)				
Yes	16 (18.2)	10 (16.9)	6 (20.7)	0.669
No	72 (81.8)	49 (83.1)	23 (79.3)	
Acute therapy, n (%)				
High-dose corticosteroids	47 (53.4)	33 (55.9)	14 (48.3)	0.093
IVIg	6 (6.8)	4 (6.8)	2 (6.9)	0.414
High-dose corticosteroids + IVIg	18 (20.5)	14 (23.7)	4 (13.8)	0.066
Inadequate treatment	17 (19.3)	8 (13.6)	9 (31.0)	
Maintenance therapy, n (%)				
Oral corticosteroids	45 (51.1)	32 (54.2)	13 (44.8)	0.020*
Oral corticosteroids + MMF	12 (13.6)	10 (16.9)	2 (6.9)	0.025*
Oral corticosteroids + RTX	11 (12.5)	9 (15.3)	2 (6.9)	0.035*
Inadequate treatment	20 (22.7)	8 (13.6)	12 (41.4)	
EDSS score at first attack, mean ± sd	3.0 ± 1.5	3.1 ± 1.7	3.0 ± 0.9	0.921
EDSS score at last follow-up, mean ± sd	2.1 ± 1.5	2.2 ± 1.7	2.0 ± 1.3	0.544
Rate of EDSS change after treatment, mean ± sd, %	31.6 ± 35.9	28.7 ± 35.1	37.4 ± 37.4	0.283

Data are presented as mean ± SD or number (percent). OR indicates odd ratio; CI, confidence interval; ADEM, acute disseminated encephalomyelitis; CSF, cerebro-spinal fluid; MOG, myelin oligodendrocyte glycoprotein; IVIg, intravenous immunoglobulin; MMF, mycophenolate mofetil; RTX, rituximab; and EDSS, expanded disability status scale. ^#^The serum concomitant autoantibodies included antinuclear antibody, extractable nuclear antigen antibody, double-stranded DNA antibody, antineutrophil cytoplasmic antibody, anticardiolipin antibody, Sjogren’s syndrome A antibody, Sjogren’s syndrome B antibody, rheumatoid factor, thyroglobulin antibody and thyroid peroxidase antibody. *P value < 0.05, **P value < 0.01 represent statistical significance.

29 patients (33.0%) relapsed within one year of the first onset. Comparison of the basic characteristics between relapsed and non-relapsed patients showed a higher proportion of females in the relapsed group (72.4% vs 42.4%, *P* = 0.010), a significantly increased proportion of CCE as the first phenotype in relapsed patients (27.6% vs 6.8%, *P*=0.013), and a significantly higher proportion of patients with serum MOG antibody titer ≥ 1:32 in the relapsed group (79.3% vs 49.2%, *P*=0.009). In addition, 86.4% of patients in the non-relapse group received adequate maintenance therapy, whereas only 58.6% of those who had a relapse in 1 year received similar treatment.

### Screening for risk factors for relapse of MOGAD within one year after the first onset

To contribute and validate a prediction model, the dataset was randomly divided into a training cohort and a validation cohort at an 80:20 ratio, and there were no significant differences in features between the training and validation sets (**Table 2**). As shown in **Table 3**, in the training cohort, univariate logistic regression analysis revealed associations between the risk of 1-year relapse and 5 variables: sex, CCE, serum MOG antibody titer, acute therapy and maintenance therapy (*P* < 0.1).

**Table 2:** Characteristics of patients in the training cohort and validation cohort.

Variables	Training cohort	Validation cohort	*P* value
	(N = 70)	(N = 18)	
Relapse in 1 year follow-up, n (%)			
Yes	22 (31.4)	7 (38.9)	0.573
No	48 (68.6)	11 (61.1)	
Sex, n (%)			
Female	35 (50.0)	7 (38.9)	0.400
Male	35 (50.0)	11 (61.1)	
Age at onset, mean ± sd, year	37.0 ± 15.9	38.4 ± 16.7	0.758
Time from onset to diagnosis, mean ± sd, month	9.81 ± 29.8	10.9 ± 29.2	0.885
Allergic history, n (%)			
Yes	5 (7.1)	0 (0.0)	0.243
No	65 (92.9)	18 (100.0)	
Optic neuritis, n (%)			
Yes	30 (42.9)	8 (44.4)	0.903
No	40 (57.1)	10 (55.6)	
Myelitis, n (%)			
Yes	25 (35.7)	4 (22.2)	0.277
No	45 (64.3%)	14 (77.8)	
ADEM, n (%)			
Yes	3 (4.3)	1 (5.6%)	0.818
No	67 (95.7)	17 (94.4)	
Brainstem or cerebellar deficit, n (%)			
Yes	9 (12.9)	4 (22.2)	0.318
No	61 (87.1%)	14 (77.8)	
Cerebral monofocal or polyfocal deficit, n (%)			
Yes	4 (5.7)	2 (11.1)	0.418
>No	66 (94.3)	16 (88.9)	
Cerebral cortical encephalitis, n (%)			
Yes	11 (15.7)	1 (5.6)	0.263
No	59 (84.3)	17 (94.4)	
Mixed phenotype, n (%)			
Yes	11 (15.7%)	2 (11.1)	0.624
No	59 (84.3)	16 (88.9)	
CSF pressure, mean ± sd, mmH_2_O	177.0 ± 42.7	164.0 ± 27.9	0.123
CSF leucocyte count, mean ± sd, 10^6^/L	46.7 ± 51.5	33.4 ± 35.3	0.206
CSF protein level, mean ± sd, g/L	0.92 ± 1.43	0.62 ± 0.34	0.117
Serum MOG antibody titer, n (%)			
≥ 1:32	41 (58.6)	11 (61.1)	0.845
< 1:32	29 (41.4)	7 (38.9)	
Serum concomitant autoantibodies^#^, n (%)			
Yes	12 (17.1)	4 (22.2)	0.618
No	58 (82.9)	14 (77.8)	
Acute therapy, n (%)			
High-dose corticosteroids	37 (52.9)	10 (55.6)	0.27
IVIg	4 (5.7)	2 (11.1)	
High-dose corticosteroids + IVIg	17 (24.3)	1 (5.6)	
Inadequate treatment	12 (17.1)	5 (27.8)	
Maintenance therapy, n (%)			
Oral corticosteroids	38 (54.3)	7 (38.9)	0.537
Oral corticosteroids + MMF	8 (11.4)	4 (22.2)	
Oral corticosteroids + RTX	9 (12.9)	2 (11.1)	
Inadequate treatment	15 (21.4)	5 (27.8)	
EDSS score at first attack, mean ± sd	3.0 ± 1.5	3.3 ± 1.5	0.400
EDSS score at last follow-up, mean ± sd	2.1 ± 1.5	2.1 ± 1.6	0.934
Rate of EDSS change after treatment, mean ± sd, %	30.1 ± 34.5	37.4 ± 41.4	0.496

Data are presented as mean ± SD or number (percent). ADEM, acute disseminated encephalomyelitis; CSF, cerebro-spinal fluid; MOG, myelin oligodendrocyte glycoprotein; IVIg, intravenous immunoglobulin; MMF, mycophenolate mofetil; RTX, rituximab; and EDSS, expanded disability status scale. #The serum concomitant autoantibodies included antinuclear antibody, extractable nuclear antigen antibody, double-stranded DNA antibody, antineutrophil cytoplasmic antibody, anticardiolipin antibody, Sjogren’s syndrome A antibody, Sjogren’s syndrome B antibody, rheumatoid factor, thyroglobulin antibody and thyroid peroxidase antibody.

**Table 3:** The result of univariate logistic regression in the training cohort.

Variables	OR	95% CI	*P* value
Demographic data			
Sex Female vs. Male	4.07	1.35-12.25	0.013*
Age at onset	1.00	0.97-1.04	0.866
Time from onset to diagnosis	1.01	0.99-1.02	0.442
Allergic history Yes vs. No			
Phenotype			
Optic neuritis Yes vs. No	1.17	0.42-3.22	0.766
Myelitis Yes vs. No	0.57	0.19-1.72	0.321
ADEM Yes vs. No	1.10	0.09-1.72	0.942
Brainstem or cerebellar deficits Yes vs. No	1.11	0.25-4.89	0.895
Cerebral monofocal or polyfocal deficit Yes vs. No	0.00	0.00-Inf	0.993
Cerebral cortical encephalitis Yes vs. No	5.13	1.32-20.02	0.018*
Mixed phenotype Yes vs. No	2.06	0.55-7.66	0.281
Laboratory results			
CSF pressure	1.00	0.99-1.02	0.540
CSF leucocyte count	1.00	0.99-1.01	0.963
CSF protein level	0.52	0.20-1.36	0.184
Serum MOG antibody titer ≥ 1:32 vs. < 1:32	3.40	1.08-20.70	0.036*
Serum concomitant autoantibodies^#^ Yes vs. No	1.72	0.48-6.19	0.405
Treatment			
Acute therapy			
High-dose corticosteroids vs. Inadequate treatment	0.26	0.07-1.03	0.055
IVIg vs. Inadequate treatment	0.24	0.02-3.01	0.268
High-dose corticosteroids + IVIg vs. Inadequate treatment	0.22	0.04-1.09	0.064
Maintenance therapy			
Oral corticosteroids vs. Inadequate treatment	0.17	0.05-0.61	0.006**
Oral corticosteroids + MMF vs. Inadequate treatment	0.10	0.01-1.10	0.060
Oral corticosteroids + RTX vs. Inadequate treatment	0.06	0.01-0.65	0.020*
EDSS score			
EDSS score at first attack	0.96	0.69-1.35	0.816
EDSS score at last follow-up	0.90	0.64-1.28	0.557
Rate of EDSS change after treatment	2.50	0.59-10.62	0.213

OR indicates odd ratio; CI, confidence interval; ADEM, acute disseminated encephalomyelitis; CSF, cerebro-spinal fluid; MOG, myelin oligodendrocyte glycoprotein; IVIg, intravenous immunoglobulin; MMF, mycophenolate mofetil; RTX, rituximab; and EDSS, expanded disability status scale. *P value < 0.05, **P value < 0.01 represent statistical significance.

**Figure 2:**
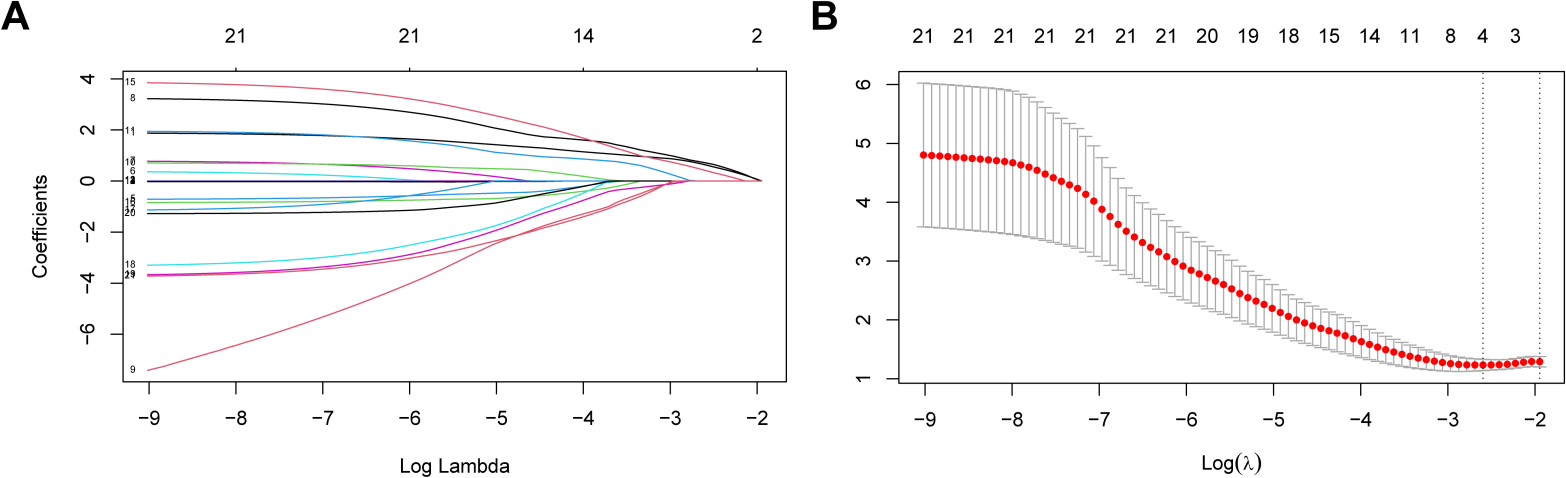
Risk factors for recurrence within 1 year in MOGAD patients selecting via LASSO regression model. **(A)** Log (lambda) value of 21 features in the LASSO model. **(B)** Parameter selection in the LASSO model uses ten-fold cross-validation through minimum criterion. Optimal lambda produces 4 nonzero coefficients. LASSO, least absolute shrinkage and selection operator.

For further screening variables, the 21 basic characteristics of MOGAD patients were included in the LASSO regression model. A total of 4 variables with non-zero coefficients were identified: female sex, CCE phenotype, serum MOG titer ≥1:32, and adequate maintenance therapy after the first attack (**Figures 2A, B**). These 4 variables were included in the multivariate logistic regression analysis. It was found that female sex, CCE phenotype, and serum MOG antibody titer ≥1:32 were independent risk factors for one-year relapse of MOGAD (*P*=0.012, 0.040, and 0.012, respectively). Whereas adequate maintenance therapy, including oral corticosteroids, oral corticosteroids + MMF, and oral corticosteroids + RTX (*P*=0.007, 0.086, and 0.006, respectively), was a protective factor against relapse (**Table 4**).

**Table 4:** Independent risk factors for MOGAD recurrence identified through multivariate logistic regression analysis.

Variables	OR	95% CI	*P* value
Sex Female vs. Male	6.10	1.48-25.15	0.012*
Cerebral cortical encephalitis	5.89	1.08-31.96	0.040*
Serum MOG antibody titer ≥ 1:32 vs. < 1:32	7.42	1.55-35.57	0.012*
Maintenance therapy			
Oral corticosteroids vs. Inadequate treatment	0.09	0.02-0.52	0.007**
Oral corticosteroids + MMF vs. Inadequate treatment	0.09	0.01-1.40	0.086
Oral corticosteroids + RTX vs. Inadequate treatment	0.02	0.00-0.34	0.006**

OR indicates odd ratio; CI, confidence interval; MOG, myelin oligodendrocyte glycoprotein; MMF, mycophenolate mofetil; and RTX, rituximab. *P value < 0.05, **P value < 0.01 represent statistical significance.

### Building a nomogram prediction model for 1-year relapse

**Figure 3:**
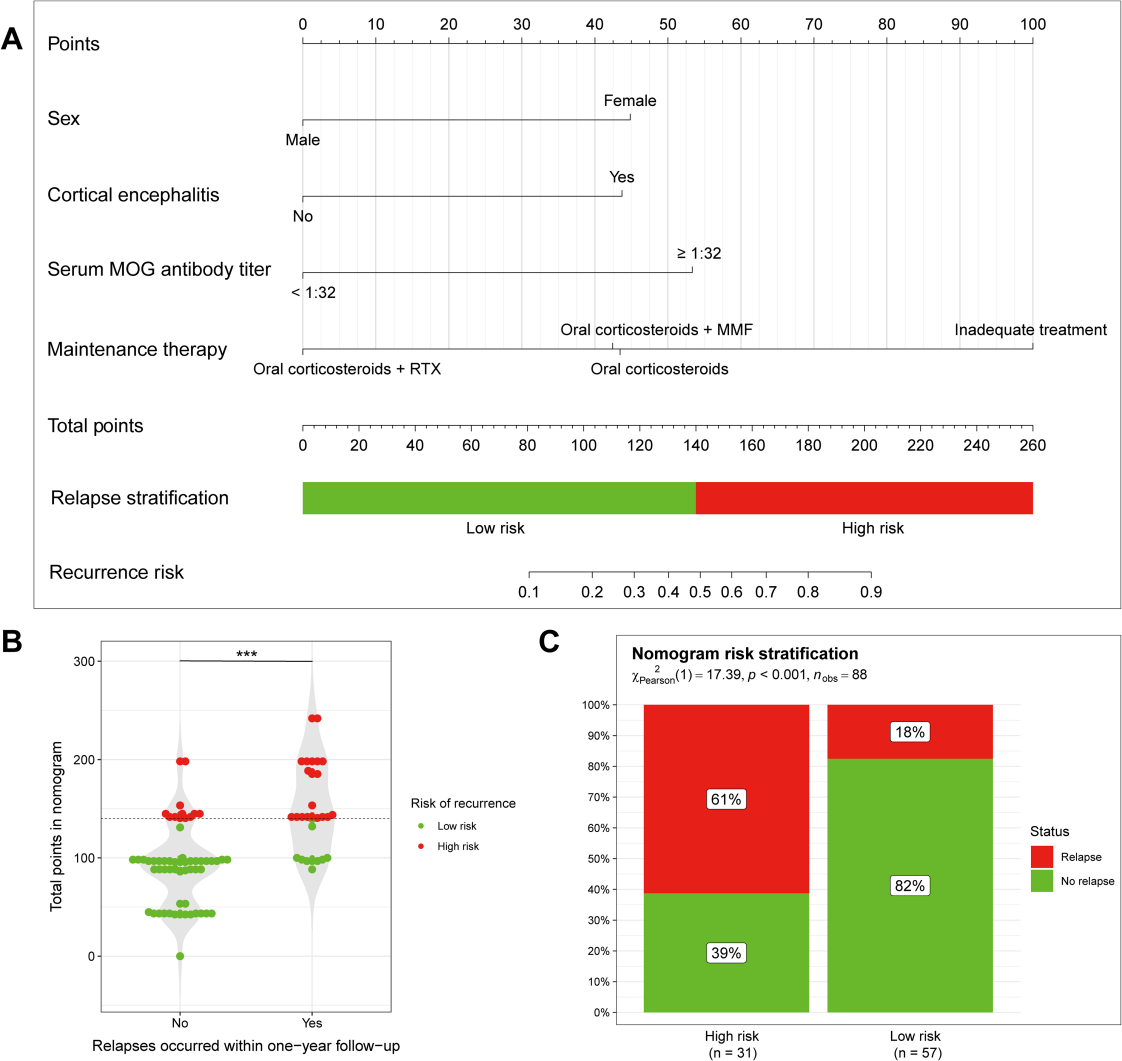
Nomogram to predict 1-year relapse in patients with MOGAD. **(A)** The nomogram model predicting disease recurrence within one year of onset, constructed using four independent risk factors. **(B, C)** Predictions of disease recurrence within one year of onset in low and high risk groups, divided according to nomogram scores. ***P < 0.005.

Incorporating with 4 independent risk factors identified through LASSO regression and multivariate logistic regression analysis, we established a nomogram model to predict MOGAD relapse within one year (**Figure 3A**). In the nomogram, each risk factor is assigned a different score. The higher the total score, the greater the likelihood of MOGAD relapse within one year. We found that the total nomogram score of patients with relapse was significantly higher than that of non-relapse patients (**Figure 3B**, *P* < 0.001). The total nomogram score for each patient in the cohort was calculated, based on the cutoff value of the ROC curve in the total nomogram score, patients were divided into high-risk and low-risk groups with a cutoff of 140 points (as shown in **Figure 3A**). Among patients in the low-risk group, only 18% relapsed within one year, while in the high-risk group, the relapse rate reached 61%. There was a significant difference in the 1-year relapse rate between the low-risk group and the high-risk group (**Figure 3C**, *P <*0.001).

### Performance evaluation of the nomogram model

**Figure 4:**
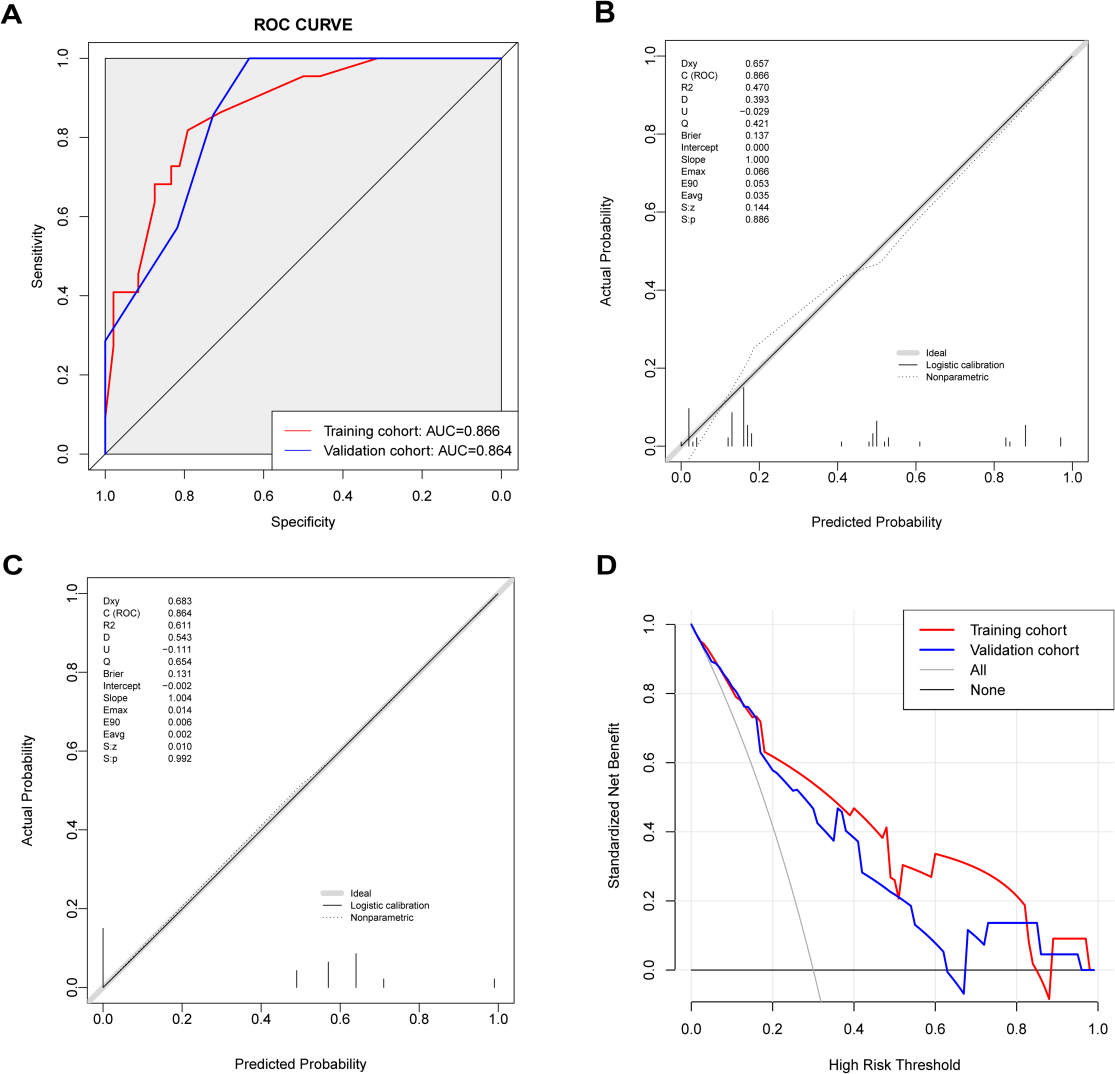
Predictive value of the nomogram model. **(A)** ROC curve of relapse in the cohort with 1-year follow-up, AUC compared between the training cohort and validation cohort. **(B)** The calibration curve indicated the probability of relapse in the training cohort with 1-year follow-up. **(C)** The calibration curve indicated the probability of relapse in the validation cohort with 1-year follow-up. **(D)** Decision curve analysis of relapse in the cohort with 1-year follow-up, compared between the training cohort and validation cohort.

The prediction model’s performance was evaluated using the AUC value (**Figure 4A**). The AUC value for the nomogram model was 0.866 (95% CI: 0.780-0.952) in the training cohort, and 0.864 (95% CI: 0.697-1.000) in the validation cohort. These results demonstrate that the nomogram model has good predictive discrimination ability in both training and validation sets. The calibration curve separately demonstrated good consistency between the relapse probability predicted by the nomogram model and the actual relapse probability in the two sets (**Figures 4B, C**). DCA curves indicated that the nomogram model had a high net benefit in identifying MOGAD one-year relapse in the two sets (**Figure 4D**).

### Using the nomogram model to predict the 2-year relapse of MOGAD

**Figure 5:**
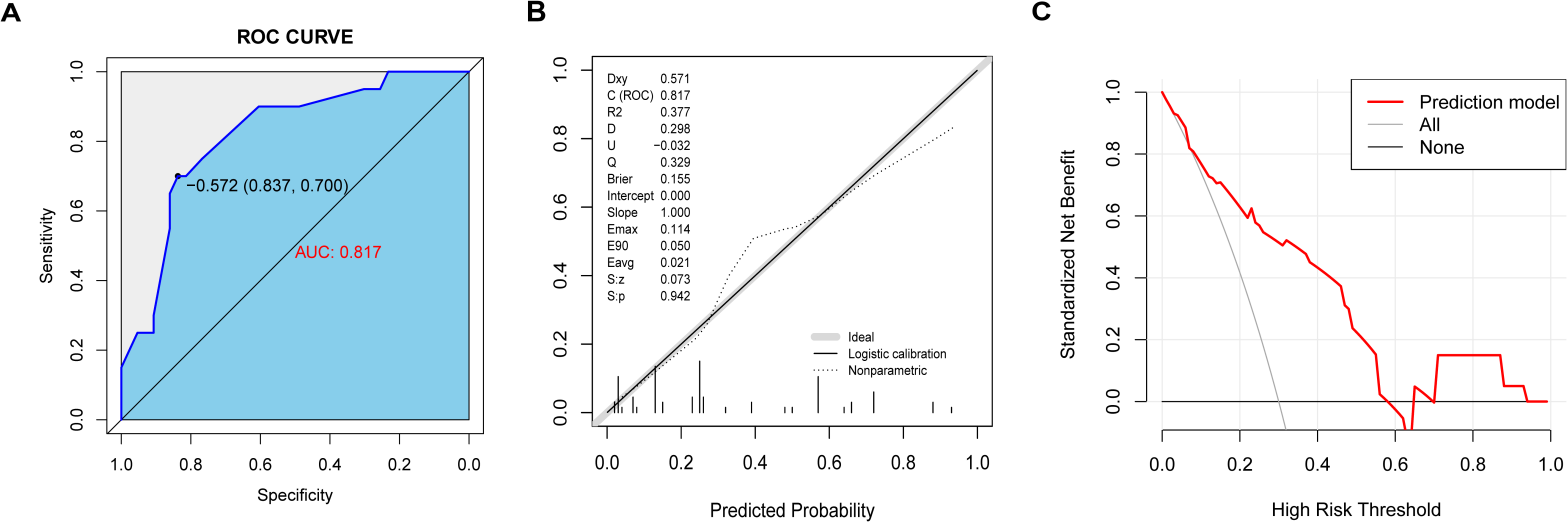
The nomogram model predicted 2-year recurrence in patients with MOGAD. **(A)** ROC curve of relapse in the cohort with 2-year follow-up. **(B)** The calibration curve indicated the probability of relapse in the cohort with 2-year follow-up. **(C)** Decision curve analysis of relapse in the cohort with 2-year follow-up.

An aggregate of 63 patients were followed up for two years after the first onset. The basic characteristics of the patients are presented in **Supplementary Table S1**. We further evaluated the nomogram model’s ability to predict a 2-year relapse of MOGAD. The AUC value of the prediction model was 0.817 (95% CI: 0.706-0.928), indicating a certain level of predictive discrimination (**Figure 5A**). The calibration curve results indicate that the predictions of the model are consistent with actual observations (**Figure 5B**). DCA shows that the model has a good net benefit in identifying 2-year relapse (**Figure 5C**).

## Discussion

Many previous studies have explored the risk factors associated with MOGAD relapse, focusing on clinical features, biomarkers, imaging features, and treatment methods. However, they primarily examine the impact of individual risk factors on relapse (13–15). Few prediction models related to disease relapse apply to clinical practice, posing a challenge to personalized clinical management. Using a single center data from the CNRID, we examined the impact of basic characteristics of MOGAD patients—such as demographic characteristics, clinical phenotype, biomarkers, treatment methods, and disease severity—on disease relapse. We identified four independent risk factors for MOGAD relapse within one year via LASSO regression and multivariate logistic regression analysis: sex, serum MOG antibody titers, phenotype, and use of maintenance therapy shortly after the first attack. Specifically, females, patients with serum MOG antibody titers ≥1:32, those with cortical encephalitis phenotype, and those who received inadequate maintenance treatment after the first onset had a higher risk of relapse. Based on these risk factors, we constructed a nomogram model to predict MOGAD relapse within one year after onset. The nomogram model demonstrated good discrimination and calibration, indicating strong predictive value. It also has a certain value in predicting MOGAD relapse two years after onset.

Based on gender statistics in this cohort, we found that the incidence ratio of MOGAD patients was nearly equal between men and women (42:46), with no obvious female predominance, which is consistent with previous studies (16, 17). However, female patients appear more prone to relapse rather than a monophasic course. Our data show that among patients who relapsed within one year of onset, the proportion of women reached 72.4%. Previous studies have also found that female MOGAD patients have a higher overall risk of relapse and an earlier first relapse (9, 14). Autoimmune diseases have been confirmed to have a gender bias, which is related to women’s stronger innate and adaptive immunity. The specific molecular mechanism remains unclear and may result from the combined effects of gender-differentiated chromosomes, hormones, and environment (18). Similar sex advantage is also observed in other central demyelinating diseases such as NMOSD with positive serum AQP4 antibodies and MS, where women have higher incidence and relapse rates (19, 20). However, the difference is that gender only affects the relapse rate of MOGAD rather than the incidence rate, suggesting that the relapse of MOGAD may follow a different sex-dependent immune pathway from the first onset. Currently, no research has investigated how sex-related factors, such as sex hormones and sex chromosomes, affect the relapse of MOGAD. Further exploration of these mechanisms may reveal new targets for preventing disease relapse.

Significant differences exist in the clinical characteristics of pediatric and adult patients with MOGAD. In pediatric cases, ADEM is the most common initial phenotype, particularly in younger children, while in adults, ON is more frequently observed as the initial phenotype. In children with recurrent MOGAD, the clinical phenotype of individual patients may evolve (21). Some studies suggest that appropriate treatment can reduce the risk of relapse, while factors such as male sex and Hispanic ethnicity are associated with a higher risk of relapse (22–24). In our study, there were only three patients aged 12 years or younger and six patients aged 18 years or younger, resulting in an insufficient number of pediatric cases to perform a detailed statistical comparison between the two groups. Therefore, further studies with larger pediatric cohorts are needed to understand better the factors contributing to the risk of relapse in children.

The clinical phenotype of MOGAD is highly heterogeneous. Consistent with previous studies, we observed that the most common phenotype is ON, accounting for nearly 50% (8, 25). However, it has no significant correlation with the first relapse within one year of onset. Myelitis is also a common phenotype, while studies have found that it may be a protective factor against MOGAD relapse (8, 9). Our data suggested that the proportion of patients with myelitis phenotype in the relapse group (20.7%) is much lower than the proportion in the non-relapse group (39.0%). However, the result is not statistically significant (*P* = 0.091) and thus was not included as a risk factor in the final prediction model. Its impact on disease relapse requires data from a larger sample size. CCE is a rare phenotype of MOGAD, with an incidence of approximately 14%. This phenotype was first described by Ogawa et al. in 2017 (12, 26, 27). Its clinical features include fever, headache, impaired consciousness, and epileptic seizures. Due to its lower incidence, the clinical characteristics and prognosis of MOGAD CCE have not yet been fully elucidated (28). Previous studies on MOGAD have focused more on the impact of common phenotypes such as ON, myelitis, and ADEM on disease relapse. In contrast, rare phenotypes such as CCE, cerebral monofocal or polyfocal deficits, and brainstem or cerebellar deficits were classified as brain and brainstem lesions, with no found correlation with relapse (8, 29). However, in this study, we found that the first-onset phenotype of CCE may be an independent risk factor for MOGAD relapse within one year. For MOGAD CCE patients, more aggressive immunotherapy may be employed to reduce the risk of relapse, and more attention should be paid to long-term follow-up to detect possible relapses early. It is worth noting that CCE is often misdiagnosed as autoimmune encephalitis or viral encephalitis initially because MOG antibody screening is not considered. A retrospective study found that the misdiagnosis rate of isolated CCE patients at the first visit was as high as 47%, with the correct diagnosis often made only after disease relapse. Delayed diagnosis may affect prognosis, suggesting that serum and CSF MOG antibody screening should be actively improved for patients with clinical or imaging features similar to CCE (30). In our cohort, one patient was found to be positive for NMDAR antibodies and experienced a relapse during follow-up. Several cases of the Myelin Oligodendrocyte Glycoprotein Antibody-Associated Disease and N-methyl-D-Aspartate Receptor Encephalitis Overlap Syndrome (MNOS) have been reported in the literature. This overlap syndrome is rare and clinically complex, with patients showing concomitant positivity for both MOG and anti-NMDAR antibodies. The pathophysiology of this condition remains unclear, and its clinical manifestations are diverse, with no standardized treatment protocols currently available. As a result, the diagnosis, management, and prognosis of this disease pose significant challenges, and its clinical course and risk of relapse are difficult to predict (31, 32).

MOG-IgG is a highly disease-specific antibody. Unlike NMOSD, where AQP4-IgG binds to AQP4 orthogonal arrays of particles (OAPs) on the surface of astrocyte foot processes and activates the downstream complement system, causing direct neurological damage, the role of MOG-IgG in MOGAD is not entirely clear. It may bind to MOG on the surface of oligodendrocytes and induce complement deposition, antibody-dependent cell-mediated cytotoxicity, and antibody-dependent cell phagocytosis, resulting in demyelinating pathological changes without damaging neuronal cells. This could explain why MOGAD presents with milder symptoms and a better prognosis compared to NMOSD (33–35). Serum MOG antibody positivity is the primary criterion for diagnosing MOGAD. Currently, the principal methods for detecting MOG-IgG titers include fixed CBA and live CBA. When using the fixed CBA method, the conformational epitope of MOG is more easily lost, potentially missing 10-15% of positive cases. Its sensitivity and specificity are lower than those of the live cell assays (12). Although the live cell method is more recommended, it has only become widely adopted in China since 2023. Therefore, this study utilized the fixed CBA method for serum MOG-IgG titers. As serum MOG antibody titers vary with disease progression, we selected patients in the acute phase of the disease and measured MOG antibody titers prior to the administration of relevant treatments (such as corticosteroids, IVIG, plasmapheresis, and immunosuppressive agents) to avoid the occurrence of false-negative results. According to the latest criteria proposed by the international MOGAD panel, a titer of ≥1:100 is considered clearly positive when using the fixed CBA method to detect serum MOG antibodies. As long as there is at least one MOGAD core clinical phenotype, a diagnosis of MOGAD can be made directly. However, a titer of ≥1:10 and <1:100 is considered low positive, and a diagnosis can only be made if there is at least one clinical or imaging support feature in addition to the core clinical phenotype (12). This study found a higher risk of relapse in the group with serum MOG-IgG titer ≥1:32. Although a titer of 1:32 is considered a low-positive antibody, implying challenges in obtaining consistent inter-laboratory results, our findings indicate that higher serum MOG antibody titers at the first onset are associated with an increased risk of relapse (36). Serum MOG-IgG is detectable at the onset of the disease and remains present for an extended period. The titer may depend on disease activity and treatment, affecting the relapse of the disease and the duration of sequelae (3, 37). A multi-center prospective study that included 116 children with MOGAD found that serum MOG antibody titers decreased after the first onset, significantly reducing the risk of disease relapse. Although there was no significant difference in antibody titers between relapsed and non-relapsed patients in the early stages, patients with a monophasic course experienced a significant drop in serum titers within one year, suggesting that lower serum MOG antibody titers may act as a protective factor against disease relapse (10). However, some studies suggest that high MOG antibody titers at onset are related to disease severity but do not reliably predict relapse course (38). This inconsistency may arise because the diagnostic criteria for the disease were only recently established, and past studies may have suffered from selection bias when enrolling patients. Additionally, the sample sizes of current MOGAD-related clinical studies are usually small. The role of MOG-IgG titers in disease activity and relapse prediction requires larger studies (12).

The reported relapse rate of MOGAD is approximately 50-70%, with a median interval from onset to relapse of about six months (27, 39). Thus, more than half of patients with relapse course will relapse within one year of the first onset. Our cohort obtained consistent data: 33.0% of MOGAD patients relapsed within one year of onset, and a total of 36.5% experienced relapse during the follow-up period. The median interval from onset to relapse was 6 months. MOGAD symptoms are milder compared to NMOSD and MS, with a lower relapse rate. However, most disability caused by the disease results from incomplete recovery after attacks or multiple relapses. Adequate acute phase and maintenance period treatments are still required (27, 39). Short-term high-dose intravenous corticosteroids treatment (1 g per day, for 3-5 days) is often used during the acute phase. MOGAD patients typically respond well to this therapy and experience significant improvement. However, patients are prone to relapse after gradually reducing and discontinuing corticosteroids (40). Our multicenter data indicated that oral corticosteroids less than 3 months increased the risk of MOGAD relapse (41). The difference is that all the patients who received oral corticosteroids in our center used corticosteroids for at least 7 months with a relatively fixed reduction plan, leading to differences from multicenter results. In cases of insufficiency in corticosteroid therapy, we implement other strategies including intravenous immunoglobulin (IVIG) (total of 0.4 g/kg for 5 days) and plasma exchange(five exchanges on alternative days) among others. However, this study did not find these acute-phase treatments valuable in predicting disease relapse. Because MOGAD has a better prognosis and fewer relapses than NMOSD, and considering the potential risks of infectious diseases and economic burdens associated with immunosuppressive therapy, medical institutions usually adopt a conservative approach toward preventive immunotherapy for the first onset until relapse occur (1, 42). In the chronic management of MOGAD to prevent relapse, the standard approach involves administering 1mg/kg orally daily for 3 months, followed by a gradual tapering over the next 3 months (40). Immunosuppressive agents may be added if necessary. Currently, commonly used immunosuppressants include mycophenolate mofetil (MMF), azathioprine (AZA), and rituximab (RTX). However, there are no randomized controlled clinical trial results, and the optimal drug dosage, duration, and associated side effects need further confirmation (42, 43). In this study, we found that adequate maintenance therapy using oral corticosteroids, steroids combined with MMF or steroids combined RTX after the first onset of the disease is a significant protective factor for MOGAD relapse. Compared to MMF treatment, RTX has demonstrated effective relapse prevention in the selection of immunosuppressive agents. Identifying patients at risk of relapse and administering appropriate immunosuppressive treatment is the focus of current management.

It is noteworthy that our study established a clinically practical nomogram prediction model with good discriminatory ability, which divides patients into high-risk and low-risk relapse groups based on the nomogram scores. Compared to individual factors, this risk-scoring model demonstrated superior performance in distinguishing relapse events within the cohort. Another interesting finding from our study is that the model also predicted relapse within 2 years, yielding a promising AUC value of 0.817. The relatively lower AUC value may be attributed to the limited sample size; however, it still suggests that the model has a certain predictive capacity for relapse over 2 years. Relapse risk can be assessed based on the patient’s clinical characteristics, guiding the screening of high-risk patients and making timely decisions on maintenance treatment. This may have positive implications for reducing the relapse probability of MOGAD patients and improving long-term prognosis, but further clinical verification is required. Our study has several limitations. A major limitation is the short follow-up period and the small sample size, which prevents a comprehensive evaluation of the impact of clinical characteristics on relapse. Another important limitation is the regional differences in patients, hospital medical levels, and the qualifications of neurologists, which are crucial in diagnosing MOGAD. Future prospective studies with larger sample sizes and longer follow-up periods are needed to confirm and expand our findings.

Overall, this study identified independent risk factors for disease relapse using demographic data, clinical manifestations, laboratory test indicators, treatment methods, and other characteristics of MOGAD patients. We established a reliable and user-friendly nomogram prediction model to assess the relapse risk of MOGAD patients, aimed at guiding personalized treatment for patients with different relapse risks.

## Data Availability

The raw data supporting the conclusions of this article will be made available by the authors, without undue reservation.

## Supplementary material

Supplementary Table 1 Baseline characteristics of MOGAD patients followed for 2 years. Data are presented as mean ± SD or number (percent). OR indicates odd ratio; CI, confidence interval; ADEM, acute disseminated encephalomyelitis; CSF, cerebro-spinal fluid; MOG, myelin oligodendrocyte glycoprotein; IVIg, intravenous immunoglobulin; MMF, mycophenolate mofetil; RTX, rituximab; and EDSS, expanded disability status scale. ^#^The serum concomitant autoantibodies included antinuclear antibody, extractable nuclear antigen antibody, double-stranded DNA antibody, antineutrophil cytoplasmic antibody, anticardiolipin antibody, Sjogren’s syndrome A antibody, Sjogren’s syndrome B antibody, rheumatoid factor, thyroglobulin antibody and thyroid peroxidase antibody. **P* value < 0.05, ***P* value < 0.01 represent statistical significance.

## References

[1] MarignierRHacohenYCobo-CalvoAPröbstelAKAktasOAlexopoulosH. Myelin-oligodendrocyte glycoprotein antibody-associated disease. Lancet Neurol. (2021) 20:762–72. doi: 10.1016/S1474-4422(21)00218-0 34418402

[2] MayerMCMeinlE. Glycoproteins as targets of autoantibodies in CNS inflammation: MOG and more. Ther Adv Neurol Disord. (2012) 5:147–59. doi: 10.1177/1756285611433772 PMC334907922590479

[3] LiXWuWHouCZengYWuWChenL. Pediatric myelin oligodendrocyte glycoprotein antibody-associated disease in southern China: analysis of 93 cases. Front Immunol. (2023) 14:1162647. doi: 10.3389/fimmu.2023.1162647 37342342 PMC10277863

[4] Cobo-CalvoARuizAMaillartEAudoinBZephirHBourreB. Clinical spectrum and prognostic value of CNS MOG autoimmunity in adults: The MOGADOR study. Neurology. (2018) 90:e1858–69. doi: 10.1212/WNL.0000000000005560 29695592

[5] ChaudhuriJRBagulJJSwathiASinghalBSReddyNCVallamKK. Myelin oligodendrocyte glycoprotein antibody-associated disease presenting as intracranial hypertension: A case report. Neurol Neuroimmunol Neuroinflammation. (2022) 9:e200020. doi: 10.1212/NXI.0000000000200020 PMC958146036261298

[6] HorJYFujiharaK. Epidemiology of myelin oligodendrocyte glycoprotein antibody-associated disease: a review of prevalence and incidence worldwide. Front Neurol. (2023) 14:1260358. doi: 10.3389/fneur.2023.1260358 37789888 PMC10542411

[7] RedenbaughVChiaNHCacciaguerraLMcCombeJATillemaJMChenJJ. Comparison of MRI T2-lesion evolution in pediatric MOGAD, NMOSD, and MS. Mult Scler Houndmills Basingstoke Engl. (2023) 29:799–808. doi: 10.1177/13524585231166834 PMC1062658137218499

[8] SatukijchaiCMarianoRMessinaSSaMWoodhallMRRobertsonNP. Factors associated with relapse and treatment of myelin oligodendrocyte glycoprotein antibody-associated disease in the United Kingdom. JAMA Netw Open. (2022) 5:e2142780. doi: 10.1001/jamanetworkopen.2021.42780 35006246 PMC8749481

[9] HudaSWhittamDJacksonRKarthikeayanVKellyPLinakerS. Predictors of relapse in MOG antibody associated disease: a cohort study. BMJ Open. (2021) 11:e055392. doi: 10.1136/bmjopen-2021-055392 PMC863428034848526

[10] WendelEMThonkeHSBertoliniABaumannMBlaschekAMerkenschlagerA. Temporal dynamics of MOG antibodies in children with acquired demyelinating syndrome. Neurol Neuroimmunol Neuroinflammation. (2022) 9 (6):e200035. doi: 10.1212/NXI.0000000000200035 PMC956204436229191

[11] ChenJJFlanaganEPBhattiMTJitprapaikulsanJDubeyDLopez ChiribogaASS. Steroid-sparing maintenance immunotherapy for MOG-IgG associated disorder. Neurology. (2020) 95:e111–20. doi: 10.1212/WNL.0000000000009758 PMC745532232554760

[12] BanwellBBennettJLMarignierRKimHJBrilotFFlanaganEP. Diagnosis of myelin oligodendrocyte glycoprotein antibody-associated disease: International MOGAD Panel proposed criteria. Lancet Neurol. (2023) 22:268–82. doi: 10.1016/S1474-4422(22)00431-8 36706773

[13] NevesALCabralASerrãoCOliveiraDSAlvesJAlvesJM. Blood neutrophils, oligoclonal bands and bridging corticosteroids as predictive factors for MOGAD course: Insights from a multicentric Portuguese cohort. Mult Scler Relat Disord. (2024) 92:105935. doi: 10.1016/j.msard.2024.105935 39427600

[14] VirupakshaiahASchoepsVARaceJWaltzMSharayahSNasrZ. Predictors of a relapsing course in myelin oligodendrocyte glycoprotein antibody-associated disease. J Neurol Neurosurg Psychiatry. (2024). 96(1):68–75. doi: 10.1136/jnnp-2024-333464 PMC1165225538964848

[15] DeschampsRGuillaumeJCironJAudoinBRuetAMaillartE. Early maintenance treatment initiation and relapse risk mitigation after a first event of MOGAD in adults: the MOGADOR2 study. Neurology. (2024) 103:e209624. doi: 10.1212/WNL.0000000000209624 38991174

[16] SechiECacciaguerraLChenJJMariottoSFaddaGDinotoA. Myelin oligodendrocyte glycoprotein antibody-associated disease (MOGAD): A review of clinical and MRI features, diagnosis, and management. Front Neurol. (2022) 13:885218. doi: 10.3389/fneur.2022.885218 35785363 PMC9247462

[17] DuchowABellmann-StroblJFriedeTAktasOAngstwurmKAyzenbergI. Time to disability milestones and annualized relapse rates in NMOSD and MOGAD. Ann Neurol. (2024) 95:720–32. doi: 10.1002/ana.26858 38086777

[18] XingEBilliACGudjonssonJE. Sex bias and autoimmune diseases. J Invest Dermatol. (2022) 142:857–66. doi: 10.1016/j.jid.2021.06.008 34362556

[19] ArnettSChewSHLeitnerUHorJYPaulFYeamanMR. Sex ratio and age of onset in AQP4 antibody-associated NMOSD: a review and meta-analysis. J Neurol. (2024) 271:4794–812. doi: 10.1007/s00415-024-12452-8 PMC1131950338958756

[20] PappVMagyariMAktasOBergerTBroadleySACabreP. Worldwide incidence and prevalence of neuromyelitis optica: A systematic review. Neurology. (2021) 96:59–77. doi: 10.1212/WNL.0000000000011153 33310876 PMC7905781

[21] SantoroJDBeukelmanTHemingwayCHokkanenSRKTennigkeitFChitnisT. Attack phenotypes and disease course in pediatric MOGAD. Ann Clin Transl Neurol. (2023) 10:672–85. doi: 10.1002/acn3.51759 PMC1018773137000895

[22] NosadiniMEyreMGiacominiTValerianiMDella CorteMPraticòAD. Early immunotherapy and longer corticosteroid treatment are associated with lower risk of relapsing disease course in pediatric MOGAD. Neurol Neuroimmunol Neuroinflamm. (2023) 10:e200065. doi: 10.1212/NXI.0000000000200065 36446614 PMC9709714

[23] BrownAMSridharAGliksmanFThomasFPPandeyKS. Clinical characteristics of pediatric and adult myelin oligodendrocyte antibody-associated disease (MOGAD): A single-center study in the Northeast. Mult Scler Relat Disord. (2024) 92:105950. doi: 10.1016/j.msard.2024.105950 39541821

[24] MartinKSrikanthPKanwarAFalardeauJPetterssonDYadavV. Clinical and radiographic features of a cohort of adult and pediatric subjects in the Pacific Northwest with myelin oligodendrocyte glycoprotein antibody-associated disease (MOGAD). Mult Scler Relat Disord. (2024) 81:105130. doi: 10.1016/j.msard.2023.105130 37979410 PMC10842716

[25] TanakaKKezukaTIshikawaHTanakaMSakimuraKAbeM. Pathogenesis, clinical features, and treatment of patients with myelin oligodendrocyte glycoprotein (MOG) autoantibody-associated disorders focusing on optic neuritis with consideration of autoantibody-binding sites: A review. Int J Mol Sci. (2023) 24:13368. doi: 10.3390/ijms241713368 37686172 PMC10488293

[26] OgawaRNakashimaITakahashiTKanekoKAkaishiTTakaiY. MOG antibody-positive, benign, unilateral, cerebral cortical encephalitis with epilepsy. Neurol Neuroimmunol Neuroinflammation. (2017) 4:e322. doi: 10.1212/NXI.0000000000000322 PMC524100628105459

[27] UzawaAOertelFCMoriMPaulFKuwabaraS. NMOSD and MOGAD: an evolving disease spectrum. Nat Rev Neurol. (2024) 20(10):602–19. doi: 10.1038/s41582-024-01014-1 39271964

[28] XuMMaCDongMGuoCYangSLiuY. Two case reports and a systematic review of the literature on adult cerebral cortical encephalitis with anti-myelin oligodendrocyte glycoprotein antibody. Front Immunol. (2023) 14:1203615. doi: 10.3389/fimmu.2023.1203615 37520572 PMC10380939

[29] Cobo-CalvoARuizARollotFArrambideGDeschampsRMaillartE. Clinical features and risk of relapse in children and adults with myelin oligodendrocyte glycoprotein antibody-associated disease. Ann Neurol. (2021) 89:30–41. doi: 10.1002/ana.25909 32959427

[30] Valencia-SanchezCGuoYKreckeKNChenJJRedenbaughVMontalvoM. Cerebral cortical encephalitis in myelin oligodendrocyte glycoprotein antibody-associated disease. Ann Neurol. (2023) 93:297–302. doi: 10.1002/ana.26549 36372941 PMC10107670

[31] XieSLiBWangZ. Three cases of overlapping syndrome of myelin oligodendrocyte glycoprotein antibody disease and anti N-methyl- D-aspartate receptor encephalitis. Chin J Neuromed. (2020) 19:1164–6. doi: 10.3760/cma.j.cn115354-20200607-00457 32234141

[32] NanDZhangYHanJJinT. Clinical features and management of coexisting anti-N-methyl-D-aspartate receptor encephalitis and myelin oligodendrocyte glycoprotein antibody-associated encephalomyelitis: a case report and review of the literature. Neurol Sci Off J Ital Neurol Soc Ital Soc Clin Neurophysiol. (2021) 42:847–55. doi: 10.1007/s10072-020-04942-0 33409829

[33] MoseleyCEVirupakshaiahAForsthuberTGSteinmanLWaubantEZamvilSS. MOG CNS autoimmunity and MOGAD. Neurol Neuroimmunol Neuroinflammation. (2024) 11:e200275. doi: 10.1212/NXI.0000000000200275 PMC1125698238996203

[34] YandamuriSSFilipekBObaidAHLeleNThurmanJMMakhaniN. MOGAD patient autoantibodies induce complement, phagocytosis, and cellular cytotoxicity. JCI Insight. (2023) 8:e165373. doi: 10.1172/jci.insight.165373 37097758 PMC10393237

[35] TakaiYMisuTFujiharaKAokiM. Pathology of myelin oligodendrocyte glycoprotein antibody-associated disease: a comparison with multiple sclerosis and aquaporin 4 antibody-positive neuromyelitis optica spectrum disorders. Front Neurol. (2023) 14:1209749. doi: 10.3389/fneur.2023.1209749 37545724 PMC10400774

[36] SechiEBuciucMPittockSJChenJJFryerJPJenkinsSM. Positive predictive value of myelin oligodendrocyte glycoprotein autoantibody testing. JAMA Neurol. (2021) 78:741–6. doi: 10.1001/jamaneurol.2021.0912 PMC807704333900394

[37] HennesEMBaumannMSchandaKAnlarBBajer-KornekBBlaschekA. Prognostic relevance of MOG antibodies in children with an acquired demyelinating syndrome. Neurology. (2017) 89:900–8. doi: 10.1212/WNL.0000000000004312 28768844

[38] Cobo-CalvoASepúlvedaMd’IndyHArmanguéTRuizAMaillartE. Usefulness of MOG-antibody titres at first episode to predict the future clinical course in adults. J Neurol. (2019) 266:806–15. doi: 10.1007/s00415-018-9160-9 30607536

[39] ReindlMWatersP. Myelin oligodendrocyte glycoprotein antibodies in neurological disease. Nat Rev Neurol. (2019) 15:89–102. doi: 10.1038/s41582-018-0112-x 30559466

[40] RamanathanSMohammadSTantsisENguyenTKMerhebVFungVSC. Clinical course, therapeutic responses and outcomes in relapsing MOG antibody-associated demyelination. J Neurol Neurosurg Psychiatry. (2018) 89:127–37. doi: 10.1136/jnnp-2017-316880 PMC580033529142145

[41] XuYMengHFanMYinLSunJYaoY. A simple score (MOG-AR) to identify individuals at high risk of relapse after MOGAD attack. Neurol Neuroimmunol Neuroinflammation. (2024) 11:e200309. doi: 10.1212/NXI.0000000000200309 PMC1138595439250723

[42] WhittamDHKarthikeayanVGibbonsEKneenRChandratreSCiccarelliO. Treatment of MOG antibody associated disorders: results of an international survey. J Neurol. (2020) 267:3565–77. doi: 10.1007/s00415-020-10026-y PMC795465832623595

[43] SchiròGIaconoSSalemiGRagoneseP. The pharmacological management of myelin oligodendrocyte glycoprotein-immunoglobulin G associated disease (MOGAD): an update of the literature. Expert Rev Neurother. (2024) 24:985–96. doi: 10.1080/14737175.2024.2385941 39110029

