# Supplementary material for: The nomogram model predicts relapse risk in myelin oligodendrocyte glycoprotein antibody-associated disease: a single-center study
Source: Front Immunol. 2025 Mar 3;16:1527057. doi: 10.3389/fimmu.2025.1527057 (PMC11911489; doi:10.3389/fimmu.2025.1527057)
Supplement: Supplementary Table 1 — Baseline characteristics of MOGAD patients followed for 2 years. Data are presented as mean ± SD or number (percent). OR indicates odd ratio; CI, confidence interval; ADEM, acute disseminated encephalomyelitis; CSF, cerebro-spinal fluid; MOG, myelin oligodendrocyte glycoprotein; IVIg, intravenous immunoglobulin; MMF, mycophenolate mofetil; RTX, rituximab; and EDSS, expanded disability status scale. #The serum concomitant autoantibodies included antinuclear antibody, extractable nuclear antigen antibody, double-stranded DNA antibody, antineutrophil cytoplasmic antibody, anticardiolipin antibody, Sjogren’s syndrome A antibody, Sjogren’s syndrome B antibody, rheumatoid factor, thyroglobulin antibody and thyroid peroxidase antibody. *P value < 0.05, **P value < 0.01 represent statistical significance. [file Table1.docx]

**Table S1.** Baseline characteristics of MOGAD patients followed for 2 years.

| **Variables** | **Non-relapse in 2 year** | **Relapse in 2 year** | ***P* value** |
| --- | --- | --- | --- |
|  | **(N=40)** | **(N=23)** |  |
| Sex, n (%) |  |  |  |
| Female | 20 (50.0) | 14 (60.9) | 0.406 |
| Male | 20 (50.0) | 9 (39.1) |  |
| Age at onset, mean ± sd, year | 36.8 ± 14.8 | 32.4 ± 17.1 | 0.290 |
| Time from onset to diagnosis, mean ± sd, month | 12.2 ± 38.0 | 16.2 ± 27.4 | 0.655 |
| Allergic history, n (%) |  |  |  |
| Yes | 2 (5.0) | 3 (13.0) | 0.272 |
| No | 38 (95.0) | 20 (87.0) |  |
| Optic neuritis, n (%) |  |  |  |
| Yes | 14 (35.0) | 12 (52.2) | 0.185 |
| No | 26 (65.0) | 11 (47.8) |  |
| Myelitis, n (%) |  |  |  |
| Yes | 15 (37.5 ) | 4 (17.4) | 0.102 |
| No | 25 (62.5) | 19 (82.6) |  |
| ADEM, n (%) |  |  |  |
| Yes | 2 (5.0) | 2 (8.7 ) | 0.567 |
| No | 38 (95.0) | 21 (91.3) |  |
| Brainstem or cerebellar deficit, n (%) |  |  |  |
| Yes | 8 (20.0) | 4 (17.4) | 0.800 |
| No | 32 (80.0) | 19 (82.6) |  |
| Cerebral monofocal or polyfocal deficit, n (%) |  |  |  |
| Yes | 4 (10.0) | 0 (0.0) | 0.993 |
| No | 36 (90.0) | 23 (100.0) |  |
| Cerebral cortical encephalitis, n (%) |  |  |  |
| Yes | 3 (7.5) | 6 (26.1) | 0.055 |
| No | 37 (92.5) | 17 (73.9) |  |
| Mixed phenotype, n (%) |  |  |  |
| Yes | 5 (12.5) | 5 (21.7) | 0.339 |
| No | 35 (87.5) | 18 (78.3) |  |
| CSF pressure, mean ± sd, mmH_2_O | 169.5 ± 38.9 | 185.9 ± 40.5 | 0.125 |
| CSF leucocyte count, mean ± sd, 10^6^/L | 38.9 ± 44.5 | 40.7 ± 38.4 | 0.869 |
| CSF protein level, mean±sd，g/L | 1.0 ± 1.8 | 0.5 ± 0.3 | 0.084 |
| Serum MOG antibody titer, n (%) |  |  |  |
| ≥ 1:32 | 18 (45.0) | 17 (73.9) | 0.030* |
| < 1:32 | 22 (55.0) | 6 (26.1) |  |
| Serum concomitant autoantibodies^#^, n (%) |  |  |  |
| Yes | 6 (15.0) | 5 (21.7) | 0.500 |
| No | 34 (85.0) | 18 (78.3) |  |
| Acute therapy, n (%) |  |  |  |
| High-dose corticosteroids | 18 (45.0) | 13 (56.5) | 0.840 |
| IVIg | 4 (10.0) | 1 (4.3) | 0.344 |
| High-dose corticosteroids + IVIg | 12 (30.0) | 4 (17.4) | 0.273 |
| Inadequate treatment | 6 (15.0) | 5 (21.7) |  |
| Maintenance therapy, n (%) |  |  |  |
| Oral corticosteroids | 23 (57.5) | 15 (65.2) | 0.673 |
| Oral corticosteroids + MMF | 6 (15.0) | 1 (4.3) | 0.178 |
| Oral corticosteroids + RTX | 4 (10.0) | 1 (4.3) | 0.324 |
| Inadequate treatment | 7 (17.5) | 6 (26.1) |  |
| EDSS score at first attack, mean ± sd | 3.2 ± 1.9 | 2.8 ± 0.8 | 0.409 |
| EDSS score at last follow-up, mean ± sd | 2.2 ± 1.8 | 1.8 ± 1.2 | 0.320 |
| Rate of EDSS change after treatment, mean ± sd, % | 29.7 ±33.3 | 39.6 ±37.2 | 0.279 |

Data are presented as mean ± SD or number (percent). OR indicates odd ratio; CI, confidence interval; ADEM, acute disseminated encephalomyelitis; CSF, cerebro-spinal fluid; MOG, myelin oligodendrocyte glycoprotein; IVIg, intravenous immunoglobulin; MMF, mycophenolate mofetil; RTX, rituximab; and EDSS, expanded disability status scale. ^#^The serum concomitant autoantibodies included antinuclear antibody, extractable nuclear antigen antibody, double-stranded DNA antibody, antineutrophil cytoplasmic antibody, anticardiolipin antibody, Sjogren's syndrome A antibody, Sjogren's syndrome B antibody, rheumatoid factor, thyroglobulin antibody and thyroid peroxidase antibody. **P* value < 0.05, ***P* value < 0.01 represent statistical significance.
